# From engineered heart tissue to cardiac organoid

**DOI:** 10.7150/thno.67661

**Published:** 2022-03-14

**Authors:** Jaeyeaon Cho, Hyein Lee, Woongchan Rah, Hyuk Jae Chang, Young-sup Yoon

**Affiliations:** 1 Department of Medicine, Division of Cardiology, Emory University School of Medicine, Atlanta, GA 30322, USA.; 2 Severance Biomedical Science Institute, Yonsei University College of Medicine, Seoul, Republic of Korea.; 3 Division of Cardiology, Department of Medicine, Yonsei University College of Medicine, Seoul, Republic of Korea.; 4 Brain Korea 21 PLUS Project for Medical Science, Yonsei University College of Medicine, Seoul, South Korea.; 5 Karis Bio Inc., Seoul, Republic of Korea

**Keywords:** Human Pluripotent Stem Cell, Engineered Heart Tissue, Organoid, Disease Modeling, Drug Screening

## Abstract

The advent of human pluripotent stem cells (hPSCs) presented a new paradigm to employ hPSC-derived cardiomyocytes (hPSC-CMs) in drug screening and disease modeling. However, hPSC-CMs differentiated in conventional two-dimensional systems are structurally and functionally immature. Moreover, these differentiation systems generate predominantly one type of cell. Since the heart includes not only CMs but other cell types, such monolayer cultures have limitations in simulating the native heart. Accordingly, three-dimensional (3D) cardiac tissues have been developed as a better platform by including various cardiac cell types and extracellular matrices. Two advances were made for 3D cardiac tissue generation. One type is engineered heart tissues (EHTs), which are constructed by 3D cell culture of cardiac cells using an engineering technology. This system provides a convenient real-time analysis of cardiac function, as well as a precise control of the input/output flow and mechanical/electrical stimulation. The other type is cardiac organoids, which are formed through self-organization of differentiating cardiac lineage cells from hPSCs. While mature cardiac organoids are more desirable, at present only primitive forms of organoids are available. In this review, we discuss various models of hEHTs and cardiac organoids emulating the human heart, focusing on their unique features, utility, and limitations.

## 1. The need for three-dimensional culture systems for mimicking human hearts

Despite long-term and extensive investment of time and funds, about 90% of new drugs fail during phase 1 clinical trials 1. The success rate of drug candidates for cardiovascular disease and oncology is the lowest, mainly due to cardiotoxicity 2-4. Cardiotoxicity, such as drug-induced QT prolongation, can cause torsades de pointes (TdP), a rapid polymorphic ventricular tachyarrhythmia leading to sudden cardiac death 5. A broad range of drugs including anti-arrhythmics 6, 7, anti-cancer 8-10, anti-histamines 11, anti-psychotics 12, and anti-virus 13, 14 were reported to provoke cardiac side effects. Since early detection of cardiotoxicity has the foremost significance in new drug development, various model systems of drug screening have been introduced with their potential to detect cardiotoxicity. Drugs for cardiovascular diseases are usually given chronically, and therefore ensuring low toxicity is essential for preclinical development.

The ideal cells for examining cardiotoxicity are human adult cardiomyocytes (CMs) isolated from the patient's cardiac biopsy. However, the limited availability of cardiac biopsy necessitates the use of animal models or nonmyocyte cell lines. Animal models and non-CM cell lines; however, have innate drawbacks for predicting drug efficacy and cardiotoxicity 15, 16. Animal model systems are low throughput, time-consuming, and relatively expensive compared to other preclinical experiments 15. More problematic is their low predictability due to inter-species differences. A non-CM cell-line overexpressing a voltage-dependent potassium channel (hERG) was widely used as a drug testing system to evaluate the risk of QT prolongation and TdP for non-cardiovascular drugs on the suggestion of the Committee for Proprietary Medicinal Products in 1997 17. However, estimation of QT prolongation in these cells could not correctly predict the response of human CMs 16. For example, verapamil and ketoconazole were predicted as potentially lethal drugs, but no cases of TdP were reported in the recipients 18, suggesting a potential attrition of valuable drugs from the market.

The emergence of human PSCs, including embryonic stem cells (hESCs) and induced PSCs (hiPSCs), presented a new opportunity for using a more physiological system for drug screening and toxicity testing because they allow generation of human CMs. hPSCs can produce an unlimited number of functional and patient-specific CMs harboring unique genetic signatures. Accumulating studies demonstrated an unprecedented variety of genetic heart disease models and corresponding drug testing results with hiPSC-CMs. Patient-derived hiPSC-CMs are known to recapitulate pathological phenotypes incurred by gene mutations and are especially useful for identifying disease-specific drug candidates and cardiotoxicity. However, hPSC-CMs differentiated in 2D monolayer culture exhibited immature CM phenotypes, restricting their utility. Excitation-contraction coupling (requiring transverse tubules (T-tubules)), positive force frequency relationship (requiring mature calcium handling), slow action potential conduction, efficient energy conversion (requiring oxidative metabolism), and the CM size were notably underdeveloped in hPSC-CMs in 2D monolayer culture 19.

Under 3D conditions, cells are cultured in a more physiological and dynamic microenvironment mimicking *in vivo* status 19. Cardiomyocytes cultured in 2D and 3D conditions showed differences in cellular morphology, expression of myofibrils, and junctional proteins 19, 20. Cells in 3D cultures exhibited less sensitivity to drugs 21 and mechanical stimuli 22, and resistance to apoptotic signaling 23, suggesting the need for 3D culture of hPSC-CMs for appropriate drug testing 24-27. Accordingly, studies have attempted to differentiate and culture hPSC-CMs in 3D culture systems 28, 29. For 3D culture, cardiomyocytes are seeded with or without other cardiac cell types embedded in a solidified gel, allowing subsequent tissue formation and mimicking the native physiological state. hPSC-CMs cultured in a 3D environment showed different electrophysiological and mechanical responses compared to those cultured in 2D systems 30, 31, and also allow non-invasive and repeated measurements of contractility 31, 32. In addition, 3D cultures of hPSC-CMs can better induce CM maturation, which is a critical challenge in regenerative medicine and drug testing. Various approaches have been developed to improve hPSC-CM maturity. Co-culture with non-cardiac cells was the most frequently used method for CM maturation^33^-^35^. Soluble factors secreted from human mesenchymal stem cells (hMSCs) co-cultured with hiPSC-CMs were employed to impact hiPSC-CM maturation 35. Extended culture periods also increased iPSC-CM maturity 36, 37. Electromechanical stress was also reported for better CM maturation. Mechanical stress 38-40 improved cardiac maturation via stretching CMs 41. Electrical stimulation 38, 39 with gradually increasing frequency over weeks also matured hiPSC-CMs. For example, physical conditioning with increasing intensity allowed hiPSC-CMs to have a transcriptionally and structurally advanced mature identity. The use of biochemical cues including changing the energy source from glucose to fatty acids 42, 43, treatment with humoral factors such as T3 44, insulin-like growth factor-1, or corticosteroids 45 were shown to induce CM maturation. Moreover, approaches combining the above methods were also developed 38. Although CM maturation characteristics such as T-tubule and Z-disks with A- and I-band were achieved through maturation of hEHT, the overall degree of maturity was far below the level of adult cardiomyocytes.

Using 3D culture platforms, two types of artificial tissues have been developed, which can emulate some of the function and structure of native heart with various sizes and shapes. Human engineered heart tissue (hEHT) is the most representative type and can be constructed by combining different types of cardiac cells and biomaterials 38, 46, 47. Another more recently developed type are human cardiac organoids, which are generated by self-organization of the differentiating hPSCs. Unlike hEHTs, organoids are induced by self-organization 48. While each model mimics a few aspects of native heart, all currently available hEHTs or organoids are dissimilar to the human heart in structure, function, and cellular components. For example, no models include native cardiac elements such as inflow and outflow tracts, each cardiac chamber, and nerves. In this review, we address the progress and applications of hEHT and cardiac organoids generated with hPSCs.

## 2. Models of human engineered heart tissue (hEHT)

The heart is a sophisticated muscular engine constantly pumping blood via the circulatory system. Efforts to emulate cardiac muscle-like tissue constructs have yielded various types of hEHTs for different purposes. Representative hEHT types include strip 26, 31, 38, 46, 49-52, ring 28, 32, patch 53-55, film 56, 57, heart-on-a-chip 58, 59, spheroid 60-65, hollow spherical chamber 66, 67 and tube types 68. Various tissue engineering technologies were employed to assemble them in three dimensions. While current hEHTs do not contain the sophisticated structure of the heart, they can represent more than one functional feature of the heart. These hEHTs are classified by production methods and characteristics, and each type has distinct advantages and disadvantages (Table 1). Representative cell culture conditions and compositions of hEHTs are summarized in Table 2.

The strip model comprised of elongated muscle fiber and shaped like a femur, is the most studied form of hEHT. The strip type is generated by the assembly of hPSC-CMs with or without supporting cardiac cells in a hydrogel including collagen, Matrigel, fibronectin or fibrin 26, 31, 38, 49. Generally, differentiated CMs on day 14 are enzymatically dissociated into single cells and mixed with supporting cells including cardiac fibroblasts. The cell mixture is suspended in a mold and maintained for more than a week for compaction, thereby forming a strip 26, 38. The blunt ends of the strip are connected to a wire or column which provides mechanical stress for cardiomyocyte maturation 38. For example, Mills et al. developed a 96-well device for functional screening of small molecules using hPSC-derived cardiac organoids (hCOs) 26. For each hCO, cardiac cells were mixed with collagen I and Matrigel and cultured in serum-free conditions in DMEM. This study demonstrated the utility of a hEHT for pro-regenerative drug development, identification of the biological mechanisms of CM maturation, and minimization of adverse side effects. The potential for mass production of hEHT from a small number of cells makes it attractive for high-throughput drug screening 49. Yumi Zhao et al. reported a heteropolar strip type with atrial and ventricular sides and chamber-specific drug responses 46. However, there is a risk of necrosis due to the lack of vasculature for diffusion, and cardiac pathological conditions such as pressure overload or myocardial infarction are not induced in this model.

The ring model is engineered by pipetting a cell mixture into circular casting molds 28, 32. The ring type differs from the strip type by a hole in the middle of the hEHT, reducing the risk of necrosis 28, 32. Additionally, the hEHT rings can be hooked around a silicon exerciser for transducing force and measuring their functions in response to electrical pulses 32, 38. The utility of the ring-type hEHTs was shown in pharmaceutical testing 32, a cryoinjury model 28, and cardiac regeneration studies 28. For example, Goldfracht et al. established ring-shaped hEHTs by differentiating hPSCs into ventricular or atrial cardiomyocytes, and then embedding these cardiomyocytes in a collagen-hydrogel to create chamber-specific, ring-shaped hEHTs 32. Ring-shaped hEHTs were transferred onto a silicon passive stretcher and cultured in IMDM Medium. The potential of this chamber-specific cardiac tissue model was demonstrated in physiologic studies, disease modeling, and drug testing. However, the maturity of the CMs was still far lower than adult human CMs 69-71.

The patch type is a flat shape of tissue and can be generated with or without scaffold. Matrigel, fibrin, or decellularized organ was used as scaffold 53-55, and temperature-sensitive dishes were utilized for scaffold-free generation of hEHT 72, 73. The patch type is mainly applied for regenerating injured hearts when transplanted onto the outer cardiac wall 53-55. For example, Gao et al. generated human cardiac muscle patches of clinically relevant dimensions (4 cm × 2 cm × 1.25 mm) by suspending cardiomyocytes, smooth-muscle cells, and endothelial cells that were differentiated from hiPSCs in a fibrin scaffold. When transplanted into a porcine infarct model, this patch-shaped tissue was shown to reduce infarct size. However, the patch type requires a large number of cells, and the risk of tissue destruction during the culture period was raised 74.

The spheroid type is a small and simple form of hEHT. Spheroids can be generated by culturing cell mixtures of hPSC-CMs, human cardiac fibroblasts, and hPSC-ECs in a non-adhesive and round-bottom 96-well plate 60-65. Archer et al. fabricated spheroids using hiPSC-CMs, human cardiac microvascular endothelial cells, and human cardiac fibroblasts (4:2:1). After 14 days, spontaneously beating microtissues were formed 63. Due to the availability of multiple spheres produced simultaneously from a relatively small number of cells, spheroids were claimed to be suitable for high-throughput drug testing 75. However, their utility is limited, since the spheroids lack vasculature and cell necrosis can occur inside spheroids larger than 250µm 76.

The heart-on-a-chip type is a module-based heart tissue connected to a microfluidic system 58, 59. Heart-on-a-chip can precisely control the microenvironment and monitor the real-time drug response of CMs 77, 78. A primitive example is a cardiac microphysiological system (MPS) developed by Maher et al. 59. By aligning CMs in the microtissue and creating separate fluid transport channels mimicking vasculature and continuous nutrient exchange, this MPS was able to keep hiPSC-derived cardiac tissue viable and functional over multiple weeks. This system allowed multiple modes of cardiac functional analysis and drug screening. However, the need for sophisticated equipment limits high-throughput drug screening. Furthermore, the microfluidic channel could absorb test drugs, preventing accurate prediction of drug response 79, 80.

The film type is a biomaterial coated sheet or low-adhesive tape with a monolayer of cardiomyocytes 56, 57. A strain sensor embedded in the thin film enables non-invasive and continuous readout. Lind et al. introduced an instrumented cardiac microphysiological device with a monolayer of hiPSC-CMs or commercially available Cor4U cell line seeded on the fibronectin coated film 124. They established high-throughput cardiotoxicity screening through flexible sensors, allowing fabrication of a multi-well platform with continuous readout of the contractile stress and beating rate. Compared to the heart-on-a-chip type, the film type can be made relatively simply with general materials in the lab 57. The forward and backward movements of the film type can be measured as the contractile capacity of the hEHT. The film type was utilized for the modeling of Barth syndrome and the cardiotoxicity drug test 57.However, CM immaturity, lack of capacity for long-term culture, and low reproducibility were claimed as disadvantages of the film models 57.

There have been attempts to generate a hollow, 3D, fluid-ejecting hEHT to mimic the native heart. The following two types have such characteristics, having a cardiac cavity enabling functional analyses such as PV loop analysis, developed pressure, cardiac output, and ejection fraction 66-68. The hollow chamber type is a fluid-pumping cardiac tissue made of either catheter or nanofibrous scaffolds 66, 67. In the catheter type, a mixture of differentiated CMs and collagen was solidified surrounding a silicon Foley catheter 67. After gel polymerization, the hollow cardiac chamber was carefully removed and incubated in a bioreactor containing cell culture medium. MacQueen et al. created tissue-engineered ventricles by using ventricle-shaped scaffold through nanofiber spinning and nanofibrous scaffolds 66. These scaffolds were incubated with fibronectin and CMs (rat ventricular CMs or hiPSC-CMs) at high density. The hollow chamber had a cavity of 500 µl of diastolic volume, or about 1/250 of the native human heart, and a very thin wall (~0.1 mm) to maintain cell viability in the diffusion-limited condition without vessels. This engineered tissue allowed measurements of multiscale *in vitro* cardiac assays including tissue coverage and alignment, calcium-transient propagation, and pressure-volume loops in the presence or absence of test compounds 66. The tube type was generated by serially wrapping custom-made tubes with hiPSC-CM cell sheets and fibroblast sheets 68. Fibrin and collagen gels were applied as glue between the attached sheets and the tube. Measurement of inner pressure was available using a catheter, and the multi-layered structure of this type is distinct from the simple combination of cells in other models. However, the tube type carries the risks of necrosis in multi-layered cell sheets without vasculature, and tube shrinkage caused by medium leakage 68.

## 3. Applications of hEHT

hEHT can present a more relevant human disease model than animal models. hEHT enables experiments that would have been impossible with hPSC-CMs in conventional 2D systems. In 2D cultures of hPSC-CMs, invasive electrophysiological experiments are the major analytical tools; however, in hEHT, noninvasive and repeated measurements of contractile force and electrophysiological activities are possible. In addition, owing to a drug resistance generally exhibited in 3D cell culture, hEHT is expected to recapitulate *in vivo* responses better than hPSC-CMs in 2D culture 81-83. Furthermore, technical advances enabled the generation of atrial 84, ventricular 67 or bipolar (atrial and ventricular ends) 46 hEHT, allowing more sophisticated modeling of chamber-specific cardiac diseases. With the aforementioned advantages, hEHT can be applied for disease modeling including genetic and non-genetic cardiac diseases, drug screening, cardiac regeneration, and cell-based cardiac pumps.

### 3.1 Disease modeling

Cardiac disease models were investigated with 2D-cultured hiPSC-CMs carrying genetic mutation(s) or induced pathological cardiac conditions 85-87. Diseased hiPSC-CMs and electrophysiological experiment systems are appropriate to investigate disorders caused by abnormal ion channel activities but are not suitable for cardiac maladaptations caused by mechano-structural problems. Recent advances in hEHT technologies enabled the investigation of various disease models overcoming the limitations of CMs cultured in 2D systems. Here, we discuss representative genetic and nongenetic cardiac diseases which can be modeled by hEHT.

#### 3.1.1 Genetic disease

Barth syndrome (BTHS) patients exhibit mutations in the TAZ gene and abnormal sarcomerogenesis in hiPSC-CMs 57. The contractile pathophysiology caused by abnormal sarcomerogenesis was investigated with the film type of hEHT by seeding purified BTHS hiPSC-CMs on a thin elastic film, which was called a muscular thin film tissue (MTF). MTF generated with BTHS hiPSC-CMs (BTHS-MTF) showed significantly lower twitch and peak systolic stress compared to controls, and introduction of modified TAZ mRNA reversed the myopathic phenotypes, demonstrating that the BTHS phenotypes were driven by a mutation in the TAZ gene 57.

Duchenne muscular dystrophy (DMD) is caused by mutations in the X-linked dystrophin gene. The majority of DMD patients eventually develop dilated cardiomyopathy (DCM) 88. A DMD disease model was made with hiPSC-CMs from a DMD patient (DMD hiPSC-CMs), and a recovered model was generated after genome editing of the dystrophin gene using CRISPR-Cas9 technology (corrected DMD) 89. hEHT generated with corrected DMD hiPSC-CMs exhibited improved contractile function over DMD hEHT but lower contractility than normal hEHT. Subsequently, corrected DMD hiPSC-CMs were mixed with DMD hiPSC-CMs in the range of 10-100% to identify the percentage of corrected CMs needed to rescue the DMD phenotype, and the gene correction required to restore the cardiac function was found to be 30 to 50% of CMs 89.

 Hypertrophic cardiomyopathy (HCM) is a polygenic disease that is strongly influenced by environmental factors and usually associated with mutations in contractile components of the sarcomere 90. Increased expression of hypertrophic markers, aberrant calcium handling, and thickening of myocardium are characteristics of HCM patients 90. hEHT models of HCM were generated with hiPSC-CMs containing BRAF 91, or PRKAG2 mutations, or electrical stimulation on hiPSC-CMs derived from a hypertension patient. BRAF encodes a serine/threonine kinase regulating the RAS/MARK pathway, which has diverse roles in cell cycle, cell growth, differentiation, and senescence 92, 93. hEHT generated with hiPSC-CMs with a BRAF mutation exhibited hypertrophic characteristics including a trend of shorter twitch duration and higher passive Young's modulus, indicating tissue stiffness. However, the pathological phenotypes were diminished only after 11 days from the hEHT formation, suggesting the need for extra stimulations or further sophisticated development of hEHT to recapitulate the cardiac hypertrophy shown in patients 94. PRKAG2 mutations can cause inherited autosomal dominant left ventricular hypertrophy 95. HCM-hEHT with a PRKAG2 mutation exhibited HCM phenotypes with increased AMPK activity and reduced adverse remodeling and arrhythmia with AMPK agonist 96. In addition, hypertrophic hEHT was generated with hiPSC-CMs derived from hypertension patients and application of electrical stimulation for up to 8 months 46. The hypertrophic hEHT showed enriched gene expression related to pathological remodeling, cardiac enlargement and dysfunction, heart failure, and cardiac hypertrophy. Chronic electrical stimulation and a long period of hEHT culture might be essential for generation of human hypertrophic heart in a dish.

Inherited arrhythmogenic syndromes, such as short QT syndrome (SQTS), were modeled using patient-specific hiPSCs. hiPSC-derived cardiac cell sheets (hiPSC-CCSs) were generated from a symptomatic SQTS patient carrying the N588K mutation in the KCNH2 gene 97 and were used as a tool for studying conduction and arrhythmogenesis. Optical mapping reported shortened APD, impaired APD-rate adaptation, abbreviated wavelength of excitation, and increased inducibility of sustained spiral waves. Phase-mapping analysis showed accelerated and stabilized rotors. Antiarrhythmic agents including quinidine, disopyramide, and sotalol were shown to rescue the arrhythmic phenotype.

#### 3.1.2 Non-genetic disease

hEHT can be used to emulate cardiac injury and cardiac response to drugs. Upon severe ischemic damage caused by coronary artery occlusion, the adult human heart undergoes pathological changes due to the limited regenerative potential of CMs, although the fetal/neonatal heart undergoes a full functional recovery through CM proliferation 98. The distinct cardiac repair responses according to CM maturation were investigated with a cryoinjury model of hEHT 28. Cryoinjury caused CM death in a localized area and high CM proliferation rather than CM hypertrophy and fibrosis, suggesting hEHT as a disease model of the immature human heart 28. This immature heart model was used for screening drugs and identifying pathways for CM proliferation, and led to the discovery of a synergistic activation of the mevalonate pathway and a cell-cycle network during CM proliferation 26.

hEHT has been proposed as a myocardial infarction (MI) model. For inducing MI-like conditions through low-oxygen supply conditions, small spheroids with a radius of ~150 µm were fabricated by the self-assembly of cardiac cells (iCell cardiomyocytes: human cardiac ventricular fibroblasts: human umbilical vein endothelial cells: human adipose-derived cells = 7:4:2:1) 62. Due to the lack of vessel formation, oxygen gradients were naturally formed in a normoxic condition (20% oxygen) and severe oxygen deprivation was created in the center of the spheroids. Necrotic core, which is considered a common problem among hEHT 99, was exacerbated by hypoxic conditions (10% oxygen). A shell of fibroblasts was found in spheroids together with cell apoptosis in the core, loss of contractile function and unsynchronized CM contractions. An anti-fibrotic reagent, JQ1 (bromodomain inhibitor) reduced the number of fibroblasts and enhanced synchronized contractions of CMs. In addition, doxorubicin exacerbated the pathological conditions 62. However, due to the severe cell death without CM regeneration and fibrosis in the core, replacing dead CMs with fibroblasts, the MI spheroid model has limitations to be addressed before deeming it an appropriate MI model.

A heart failure model was inducible in the matured hEHT by chronic catecholamine overstimulation 39, 97. A ring type of hEHT formed by a mixture of cells (hPSC-CMs and fibroblasts) and matrix (collagen and Matrigel) was induced to mature under mechanical load and conditioned media containing several growth factors including IGF and FGF. After treatment with catecholamine, hEHT exhibited contractile dysfunction, CM hypertrophy, and increased cell apoptosis 39. Heart failure model of hEHT is characterized by transcriptional profiling in 6-week engineered human myocardium (EHM) in agreement with the structural and functional data. The limitations of the heart failure model of hEHT come from the immaturity (equivalent to fetal human heart at 13 weeks of gestation).

hEHTs are also used for mimicking arrhythmias. Using linear and circular hEHTs, the dynamics of activation propagation was shown to depend on geometry 100. Linear hEHT showed the normal propagation pattern across the distal ends. However, spontaneous infinite reentrance of activation propagation was seen in the circular shape, mimicking tachycardia in a model of arrhythmogenic cardiomyopathy. Defibrillation through electrical field-stimulation reversed arrhythmias in the circular hEHT to a normal rhythm state. Consequently, the circular hEHT was suggested as an arrhythmic disease model and a screening platform for antiarrhythmic drugs. Another group also established a circular cell sheet using hiPSC-CMs for studying arrhythmogenesis via optogenetic stimulation 101. hEHTs were transduced with lentivirus expressing channelrhodopsin-2 (H134R) and stimulated by bursts of blue light separated by no pacing for 3 weeks. This optical pacing induced chronic tachycardia in the hEHTs, showing shortening of action potential duration 90 (APD90) and reduction of L-type Ca^2+^ current. This induced tachycardia was terminated by ryanodine receptor stabilization, or sodium, or hERG potassium channel inhibitor. Furthermore, arrhythmia modeled in ring-shaped atrial hEHT showed a large single circular re-entry wave propagating around the ring and multiple spiral-wave reentrant loops observed in rhythm disorders 32. Particularly, this model proposed a chamber specific, atrial-hEHT for atrial arrhythmias. Anti-arrhythmic agents vernakalant and flecainide converted the arrhythmic hEHTs to normal rhythm.

### 3.2 Drug testing and pharmacotoxicity using hEHT

hEHTs composed of major cardiac cells including CMs, fibroblasts, and endothelial cells are suggested to be an optimal drug testing platform as they can better emulate functional and structural changes of CMs in a tissue environment and have higher sensitivity to cardiotoxins compared to 2D-monolayer CMs 63. Metrics of cardiac tissue function such as contraction rate, conduction properties, and mechanical motion can be recorded in a non-invasive manner and analyzed in real-time. Chip (MPS system), strip, and spheroid types which are compatible with computer devices are generally suitable for high-throughput drug screening platforms.

Mathur et al., performed the pharmacological studies using the MPS system to test the cardiac response to four drugs (isoproterenol, metoprolol, E-4031, verapamil) 59. The motion tracking was recorded with a microscope and analyzed by automated video-optical recording. Data indicated that the half-maximal inhibitory/effective concentration values (IC50/EC50) were consistent with the data on tissue-scale references compared to cellular-scale studies. A strip type, fibrin-based hEHT in a 24-well format was established as a simple *in vitro* model for cardiac research 102, 103. Spontaneous contractions of hEHT were analyzed by automated video-optical recording. Chronotropic responses of the β-adrenergic agonist isoprenaline were observed in the presence of calcium. Also, concentration-dependent irregular beating, and reversible decreases in relaxation velocity were induced by the proarrhythmic compounds 102. Another strip type of hEHT was established with 5 commercial and 5 academic hPSC-CM lines for suitability for drug screening. Spontaneous and stimulated contractions were induced in these hEHTs for evaluating baseline contractile force, kinetics, and beating rate. Those parameters varied depending on the lines, while canonical drug responses were observed in most hEHTs. This study suggested that this hEHT may not be relevant for drug screening, while it could be used for disease modeling with the addition of isogenic controls 103. Milles et al. performed functional screening of 105 small molecules with pro-regenerative potential with their previously developed strip type platform 26. The study revealed discordance between their hEHT and conventional 2D assays for many known pro-regenerative compounds. Using their 3D hEHT, they identified two pro-proliferative small molecules that did not have detrimental effects on cardiac function. High-throughput proteomics of these two compounds revealed synergistic activation of the mevalonate pathway and a cell-cycle network, suggesting the utility of this platform for identifying biological mechanisms as well as drug screening.

hEHT was found to be useful for investigating structural changes of CMs induced by various drugs. This structural cardiotoxicity can be evaluated by morphological damage such as CM degeneration, necrosis, fibrosis, and progressive pathological changes in subcellular organelles of CMs. FDA-approved structural cardiotoxins (https://www.pharmapendium.com) were evaluated using the spheroid type of hEHT (less than 100 µm radius) generated by co-culturing of CMs, fibroblasts, and endothelial cells in an ultra-low adhesion plate 63. Structural cardiotoxicity is usually assessed for non-cardiac drugs such as anti-cancer agents. For example, lapatinib, an anti-cancer drug, was found to induce ATP depletion in endothelial cells but not in CMs and fibroblasts, suggesting that lapatinib caused the structural cardiotoxicity through the damage of endothelial cells.

### 3.3 Other applications of hEHT

Other applications of hEHTs include *in vivo* cardiac regeneration and cell based cardiac pump. A patch type of hEHT was generally used for *in vivo* cardiac regeneration. For example, this hEHT was generated with a cell mixture of human embryonic stem cell-derived CMs (hESC-CMs), human umbilical vein endothelial cells (HUVECs), and fibroblasts with 104 or without 99 a biodegradable porous scaffold. When transplanted into the injured hearts of animals, this patch, which included a mixture of CMs, ECs, and fibroblasts, substantially increased the survival of engrafted hESC-CMs, and the preexisting vessels were anastomosed with host vasculature inside the engrafted hEHT 99, 104. Generation of hEHT of a clinically relevant size was attempted through an increase in scaffold size (up to 4 x 4 cm). However, the increased scaffold size was not associated with a dramatic increase in cell number in the patch 25, 105. Regardless of the scaffold size, the total cell number was at most 8 ~ 10 million cells, suggesting the limitation of cell density in clinical application.

hEHT can also be used as a cardiac pump. Left ventricular assist devices (LVADs) could be one therapeutic option for patients with end-stage heart failure 106. However, mechanical circulatory support devices such LVADs have limitations including thrombogenicity, power transmission, and infection 107. With the development of hEHTs, a cell-based cardiac pump could be used instead of a LVAD. Such a cell-based LVAD is expected to offer a high degree of immune tolerance and create a bypass blood flow from the apex of the heart directly to the aorta 107. An optimal cell-based cardiac pump would be composed of a hollow chamber wrapped by contracting cardiac cells, one-way valves to ensure unidirectional flow, and electrical detectors and stimulators embedded as part of the device 107. The currently available cell-based pump, which is at the early developmental stage, is a tube-type hEHT (Table 1) consisting of a long hollow column wrapped by CM/fibroblast cell sheets without cardiac valves or electrical devices 68.

## 4. Human cardiac organoids

**Figure 1:**
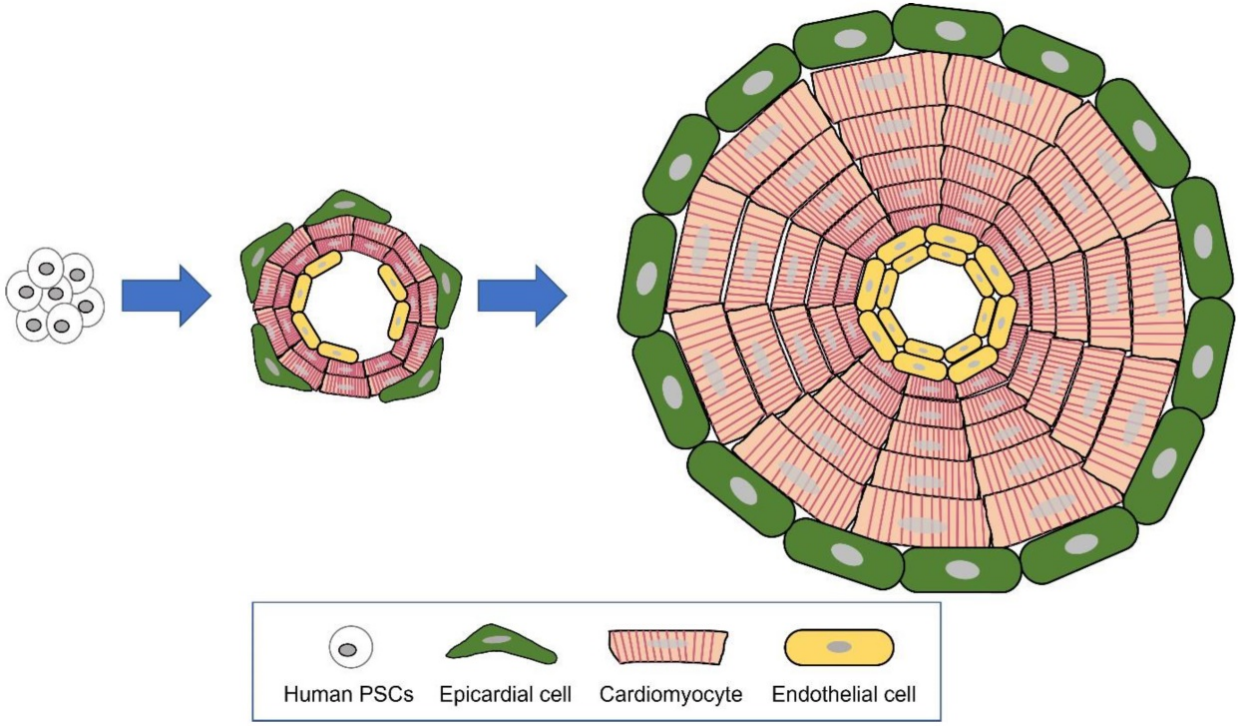
** Organoid generated through self-organization of differentiating pluripotent stem cells (PSCs)**. The major mechanisms of organogenesis are self-organization, consisting of cell sorting out (gathering of similar cell types) and spatially restricted lineage commitment.

**Figure 2:**
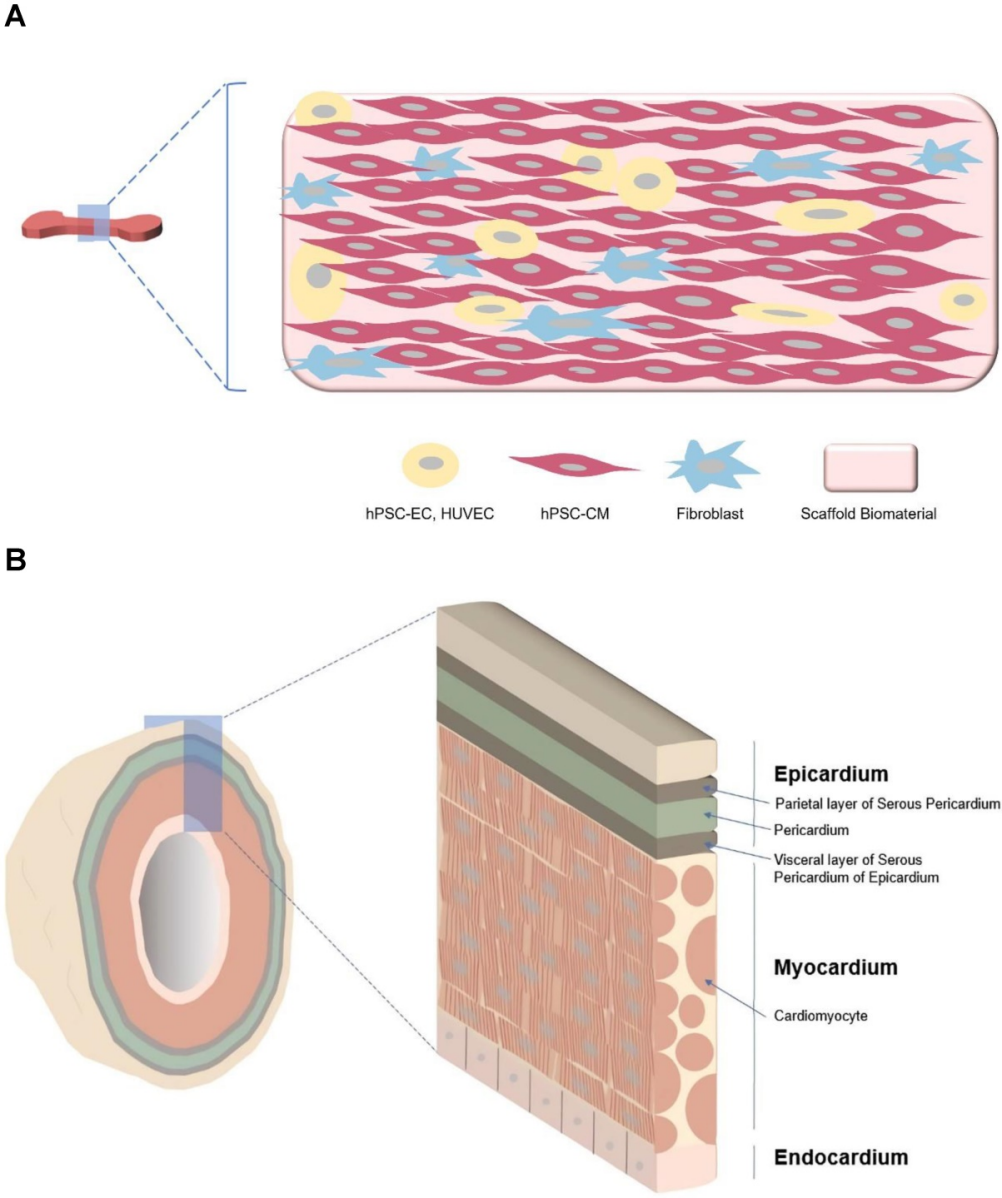
** Major differences between hEHT and cardiac organoid. A** Strip type hEHT, a most common form of hEHT. **B** Cardiac organoid resembling a native heart in cell composition (multi-cardiac cells), macro- (a hollow chamber) and micro- (multi-specialized layers composing a wall) structure, repeated systolic and diastolic contractions, and volume-pressure relationship.

An organoid is an *in vitro* 3-dimensional miniature organ which is generated with organ-specific adult stem cells or pluripotent stem cells (PSCs). According to Lancaster and Knoblich, an organoid is defined by three characteristics: 1) constitution of multiple organ-specific cell types, 2) capability to recapitulate some specific function of the organ, and 3) sorted multi-cell populations grouped together and organized similar to an organ 48. Although organoids and spheroids are both cultured in 3 dimensions and the terminology has been ambiguously used, there are distinct differences between organoids and spheroids 108, 109. Self-organization, which consists of cell sorting out and spatially restricted lineage commitment of precursor cells, is the key mechanism differentiating between organoid and spheroid 48 (Figure 1). Cell sorting out is a general capacity of cells to reorganize and classify to form structures with much the same histogenic properties. In spatially restricted lineage commitment, precursor cells give rise to more differentiated progeny by spatial constraints of the tissue and/or division orientation 48. Being self-organized, the growing organoid mimics the major processes in development. These novel tissue-patterning mechanisms offer the organoid higher complexity, having *in vivo*-like physiological features and long-term maintenance (Figure 2).

Although self-organizing organoids have been reported for almost all major organs for over a decade 110-112, human cardiac organoids were more recently developed 113, 114. Beginning with the first organoid for optic cup developed with hESCs in 2008 115, various types of organoids including brain 116, retina 117, 118, intestine 119, kidney 120-122, and liver 123 have been generated with hPSCs. For cardiac organoid-like structures, dissociated neonatal chicken 124 and rat cardiac cells 125 showed innate capacity to re-aggregate and construct a “mini-heart” with a cavity. However, a mixture of individually differentiated CMs, ECs, fibroblasts, and smooth muscle cells from human PSCs did not self-organize or grow into a “mini-heart” 114, 126, 127.

Unlike hEHTs, which use mixtures of individually differentiated cardiac cells, cardiac organoids are formed by self-organization of stem cells during cardiac differentiation (Table 3). Spontaneously contracting human cardiac organoids with cavities have been generated by several investigators. Human cardiac organoids recapitulate developmental events and model genetic malformation by specific mutations in genes or modification of the culture conditions (Table 4). Thus, cardiac organoid is a favorable model for studying cardiac development and developmental disorders. Since human cardiac organoids include vessels, 126, long-term culture is possible, which can induce more mature CMs.

Drakhlis et al. generated heart-forming organoids (HFO) with hPSC aggregates embedded in Matrigel via biphasic WNT pathway modulation 128. HFOs are composed of a myocardial layer lined by endocardial-like cells surrounded by septum-transversum-like anlagen. HFOs also possess spatially and molecularly distinct anterior versus posterior foregut endoderm tissues and a vascular network. The architecture of HFOs recapitulates early cardiomyogenesis, which requires an interplay between cardiac mesoderm and foregut endoderm. Disease models using HFOs with a NKX2.5-knockout (KO) hESC reporter line showed a phenotype previously observed in the same gene knockout mice (less organized, larger cardiomyocytes, and decreased cell adhesion).

Hofbauer et al. established hPSC-derived self-organizing cardioids that intrinsically specify, pattern, and morph into chamber-like structures containing a cavity, reminiscent of the early human left ventricular heart chamber. Beating organoids with small homogeneous sizes were developed within the dish without exogenous ECM and using a high-throughput approach to reach optimal signaling conditions 126. The average diameter of cardioids at CM specification (day 5.5~7.5) was about 2,000 μm. The cardioid corresponded to the first heart field lineage and the early stages of cardiogenesis. The study further demonstrated that this cardioid can model mechanisms underlying development of the three major components of cardiac architecture CMs, endothelial layers and epicardial lining as well as response to cardiac injury (cryoinjury). While this paper explores the potential for generating an organoid with a cavity, events at a late developmental stage are lacking, such as cardiac structure formation, maturity of cardiac cells, or compaction of myocardium. To examine the response to cryoinjury, cardioids were cultured with epicardial cells; however, co-cultures do not meet the concept of organoid as it is not an intrinsic organogenesis process.

Lewis-Israeli et al. generated human heart organoids (hHOs) using a two-step canonical Wnt signaling modulation strategy using growth factors and chemical inhibitors 129. hHOs mimic human cardiac development and are similar to age-matched fetal heart tissues at the transcriptional, cellular, and structural levels. hHOs develop internal cardiac chambers, with multiple lineage cells having regional identities reminiscent of the heart fields and the atrial and ventricular chambers, epicardium, endocardium, and coronary vasculature. These hHOs were shown to model congenital heart disease induced by pregestational diabetes, suggesting the utility of this model for emulating the effects of complex metabolic disorders on cardiac development. However, the maturation level of hHO is restricted to embryonic fetal hearts, and hHOs tend to deviate from their normal developmental pathway as a function of time, becoming less relevant over time.

Song et al. fabricated cardiac mesoderm cell-derived cardiac organoids (CMC-COs) and CM-derived COs (CM-COs) to investigate the effect of differential self-organizing capacity of mesoderm-derived cells or CMs on maturation of CMs in organoids 130. hPSC-derived CMCs and -CMs were dissociated at day 4 or 11 of cardiac differentiation and then plated on poly (2-hydroxyethyl methacrylate) (poly-HEMA)-coated plates. CMC-COs exhibited structural differences compared with CM-COs such as more organized sarcomere structures and mitochondria, well-arranged T-tubule structures, evenly distributed intercalated discs, and increased expression of ventricular CM and junctional markers. CMC-COs showed mature ventricular-like function including faster motion vector speed, decreased beats per min, increased peak-to-peak duration, and prolonged APDs. This study further revealed that LEFTY-PITX2 signaling plays a crucial role for CM maturation and specification into ventricular-like CMs.

Recently, Silva et al. used hiPSCs to produce multi lineage organoids that recapitulate cooperative cardiac and gut development and maturation 131. Mesendoderm progenitor aggregates were differentiated in cardiac-permissive medium supplemented with ascorbic acid. Multilineage organoid progenitor cells have a transcriptomic profile that supports the co-development of cardiac and gut tissues. The cardiac and gut organoid was maintained for a long period of time (>1 year) in culture and exhibited a millimeter size-scale and improved physiological maturation of cardiac tissue. However, there are technological hurdles to image large size organoids at later stages of culture due to significant light scattering during light microscopy imaging.

## 5. Remaining challenges and future perspectives

While a number of papers claimed a hEHT model as a cardiac organoid, those hEHTs fell short of the original definition of an “organoid”. This confusion might have arisen from a misunderstanding of the main defining principle of organoids, i.e., self-organization of cells. Thus, there is a need for a uniform definition in this field. Our review attempts to distinguish these two entities by the criteria of “self-organization” of stem cells.

Various methods of hEHT generation were reported and their utility was highlighted for drug screening, disease modeling, and cardiac regeneration. In general, hEHTs are constructed by combining cardiac cells including CMs with natural or artificial matrix using engineering technologies. hEHTs do not mimic the gross structure of the heart and represent one or several functions of the heart (Table 1). Since each model has a specific merit, it would be better to understand their utility in the context of a specific purpose. Among them, the main utility focuses on drug screening and disease modeling. The advantage of hEHTs over pure CMs for such purposes is their similarity to the *in vivo* environment and their superiority for CM maturation. To guarantee validity of drug testing and disease modeling when using *in vitro* cell or tissue systems, use of mature CMs is crucial. While CM maturity is improved with hEHTs, most hEHTs fall short of reaching the neonatal state of the heart. To mature the CMs in hEHTs, not one but a combination of biochemical, electrical, mechanical, and tissue engineering technologies would be required. Moreover, most hEHTs lack vasculature, limiting the size and culture period of hEHT without cell death. This is important because CM maturation needs long-term culture. For cardiac regeneration, a patch type hEHT has been widely used. At present, most patches do not show scalability and have a low cell density per unit volume. Cardiac cells need to have close or direct contact with neighbors to function properly; however, engineering technologies to organize cells at high density and of sufficient size (a few centimeters in length and width) without inducing cell necrosis are undeveloped. A minimum of 1-10 billion cells were suggested for clinically meaningful regeneration purposes 132. Optimal technologies for hEHTs need to organize a large number of hPSC-derived cardiac cells at high density, with vasculature, into a micro-structure mimicking the heart.

Such challenges have been in part addressed by organoids generated from differentiated hPSCs. The most sophisticated currently developed cardiac organoids take primitive heart forms having a cavity and cardiac walls composed of thin layers of epicardial cells and cardiomyocytes, with or without vasculature (Table 4). At the structural level, cardiac organoids better represent the structure of the native heart than hEHTs. Despite their structural closeness to the native heart, no cardiac organoids were shown to generate flow by contraction, although one of the major functions of the heart is its pumping function. Thus far, cardiac organoids have been mostly applied to cardiac developmental studies and disease modeling, while one study reported their utility for studying the response to cryoinjury 126. Due to the difficulties of monitoring the function of organoids, which are complex in structure, their utility for drug testing has yet to be reported. At present, these self-organized cardiac organoid models lack a system to monitor or control their function in real-time. In addition, the maturity of CMs in the organoids is at best at the level of fetal heart. Therefore, it would help to incorporate bioengineering technologies to equip monitoring and further induce CM maturation. For example, a bioengineering technology to control the flow in and out of organoids would help regulate drug concentrations in drug testing systems. In addition, accessibility to two-way cameras to monitor voltage/calcium transients or ultrasound probes for echocardiography would enhance the utility of organoids. A combination of self-organizing cardiac organoids together with controllable bioengineering technologies could advance and expand their utility for drug screening, disease modeling, developmental studies, cardiac regeneration, and cardiac pump. In the future, a more physiological cardiac organoid emulating native heart is required, which would be a multilayered organ having thick myocardium together with endothelium and epithelium, atrial and ventricular chambers with their own cavities, one-way valves between chambers to ensure unidirectional flow inside the organ, vascularization throughout the organoid, and innervation.

## Figures and Tables

**Table 1:** Various types of hEHTs

Type	Shape	Generation	Characteristics	Applications
Strip	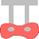	Compaction of cells and hydrogel around two parallel wires or posts^26^, ^31^, ^38^, ^46^, ^49^-^52^	Drug testing; No vasculature; Limited diffusion	Drug screening
Ring	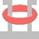	Condensation of hydrogel with cells in circular casting mold and transfer onto silicon passive stretcher^28^, ^32^	Applicable for electrical pacing studies; No vasculature; Arrhythmogenic	Disease Modeling; Drug screening
Patch	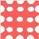	Accumulation of cell layers on coated plates^53^-^55^	Regenerative therapies; Higher number of cells required; Low throughput; Risk of breaking; Unequal distribution of the cells	Cardiac Regeneration; Disease modeling
Film	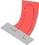	Seeding of cells onto coated film 56, 57	Limited cell-cell and cell-matrix interactions; Embedded electrode	Disease modeling
Microfluidic Chips	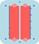	Seeding of cells onto chip (MPS)^58^, ^59^	Easy manipulation of the microenvironment; Realtime on-chip analysis; Limited cell-cell and cell-matrix interactions; Limited force measurement; Requiring multi-step fabrication; Unintended drug absorption	Drug screening
Spheroid	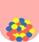	Assembly of cell mixture and hydrogel^60^-^65^	No requirement for expensive instruments; High-throughput drug testing; Necrosis in the core (>250) (limitation of diffusion) Not feasible for electromechanical stimulation	Disease modeling; Drug screening
Chamber	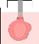	Cell seeding in agarose mold around balloon Foley catheter^66^, ^67^	Recapitulating 3D structure of the heart; Low throughput; No chamber specification; Catheter related complications	Disease modeling; Drug screening
Tube	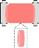	Wrapping of cell sheets (CM, fibroblasts) around a hollow column^68^	Emulating the multi-layered cardiac wall; Low throughput; Medium leakage leading to the shrinkage of the tube; Requiring high percent of fibroblasts for wall stiffness	Cell-based cardiac pump

**Table 2:** Representative cell compositions and culture conditions of hEHTs

Type	Paper	Cell composition	Culture condition
Strip	Mills et al. 26	hESC-CMs (5 × 10^4^)	α-MEM with 10% fetal bovine serum (FBS)
			bovine collagen I and Matrigel
			electrical stimulation
	Huebsch et al. 49	hiPSC-CMs: fibroblast final density: 2 x 10^7^ cells/mL	maintained in EB20 media
	Tulloch et al. 40	hESC-CMs (2 x 10^6^) HUVEC (1 x 10^6^) MSCs or MEFs (1 x 10^6^)	RPMI medium with B27 supplement
			collagen type I
			uniaxial mechanical stress conditioning
	Ronaldson-Bouchard et al. 38	hiPSC-CMs 75% Fibroblast 25%	DMEM supplemented with 10% FBS
			human fibrinogen and human thrombin
			electrical stimulation
	Zhao et al. 46	hESC-CMs: cardiac fibroblasts atrial 10:1.5 ventricle 10:1 final density: 5.75 x 10^4^ cells/mL	DMEM, 10% FBS
			rat tail collagen and Matrigel
			electrical stimulation
Ring	Goldfracht et al. 32	hESC-derived atrial/ventricular cells (2 x 10^6^)	IMDM
			bovine collagen
			electrical stimulation
Patch	Gao et al.^105^	hPSC-CMs: SMCs: ECs (2:1:1)	DMEM containing 10% fetal calf serum, B27+, Ɛ-aminocaproic acid, and ROCK inhibitor
			fibrinogen, Matrigel, and thrombin solution
			mechanical stimulation
	Ye et al.^133^	hiPSC-CMs (3.5 x 10^5^) hiPSC-ECs (4 x 10^5^) hiPSC-SMCs (3.5 x 10^5^)	1:1 mixture of medium collected from hiPSC-ECs and hiPSC-SMCs that had been cultured in serum- and glucose-free MEM medium
			fibrin with thrombin
	Stevens et al.^99^	hESC-CM (2 x 10^6^) HUVEC (2 x 10^6^) MEF (1 x 10^6^)	RPMI medium with B27 supplement
			electrical pacing
	Amano et al.^55^	hiPSC-CMs (1 x 10^6^) cardiac microvascular endothelial cells (1 x 10^6^) cardiac fibroblasts (1 x 10^6^)	DMEM
			fibronectin and gelatin
Film	Lind et al.^134^	hiPSC-CMs (2.2 x 10^6^) NRVMs (1 x 10^6^)	M199 containing 2% FBS
			fibronectin
			electrical point stimulation
Chip	Matuhr et al.^59^	hiPSC-CMs (4~5 × 10^5^)	maintained in EB20 media
Spheroid	Archer et al.^63^	hiPSC-CMs: primary human cardiac microvascular endothelial cells: primary human cardiac fibroblasts (4:2:1) final density: 8 x 10^4^ cell/mL	cardiomyocyte maintenance medium: endothelial basal medium 2 (1:1)
Chamber	MacQueen et al. 66	hiPSC-CMs (1 x 10^6^)	M199 plus vitamin B12
			human fibronectin
Tubular	Tsuruyama et al. 68	hiPSC-CMs (1 x 10^6^) NHDF (1 x 10^6^)	DMEM
			fibrin and collagen
			electrical stimulation

**Table 3:** Comparisons of hEHT and cardiac organoid

	hEHT	Cardiac organoid
Cell source	hPSC-CM hPSC-CM with non-cardiac cells	Differentiating hPSC
Use of a mold during tissue formation	O	X
Connection with an instrument for real-time assessment	O (except spheroid)	X
Developmental study	Unfeasible	Feasible
Functional assay	- Contractility/force generation - Intraventricular pressure/volume - Action potential - calcium transient	- Contractility/beating rate - Action potential - Calcium transients
Applications	- Cardiotoxicity test - Drug efficacy test - Disease modeling - Cardiac regeneration - Cardiac pump	- Developmental studies - Disease modeling ▪ congenital cardiac defect ▪ cryoinjury

**Table 4:** Human Cardiac Organoids

Model	Formation	Characteristics	Applications	Multilayered cardiac chamber	
				Chamber	Sponta-neous beating
Heart-forming organoids (HFOs)	Differentiating hPSC aggregates embedded in Matrigel via biphasic WNT pathway modulation 128	Stage: pre-heart tube-like stage at 2-3 weeks of human gestation	Cardiac development, cardiac malformation in HFOs generated with *NKX2.5*-knockout hESCs	X	
		Anterior-posterior endoderm patterning, EC-lined vessel-like structures, distinct foregut endoderm tissues		X	O
Cardioid	hPSCs differentiated with chemically defined medium 126	Stage: early human ventricular heart chamber	Cardiac development, Cryoinjury to model myocardial infarction	O	
		Self-organization of CMs and endothelial cells		O	O
		Engineered heart organoid; Addition of epicardial cells			
Human heart organoid (hHO)	Differentiation of hPSC-EB with three-step WNT signaling modulation^129^	Stage: embryonic fetal heart	Cardiac development, Modulation of glucose and insulin level to examine the effect of pregestational diabetes on cardiac development	X	
		Containing major cardiac cells without spatially restricted lineage commitment; Vascularization		O	O
CMC or CM -derived cardiac organoid (CMC or CM-COs)	hPSC-CMs cultured with B27 without insulin (CMC-COs)	Stage: early cardiac developmental stage	Cardiac development	X	
	B27 without vitamin A (CM-COs) +thiazovivin and repeated size selection(>70µm) 130	Spatially and metabolically matured CMC-COs compared to CM-COs		X	O
Multi lineage organoid	hiPSCs-derived mesendoderm progenitor aggregates differentiated with cardiac-permissive medium + ascorbic acid 131	Stage: early human embryonic heart (specifically atrial tissue)	Cardiac development	O	
		Formation of the epicardial layer and primitive endoderm epithelial cystic structure		O	O
		Extensive tissue growth during >1 year culture			

HFO: heart-forming organoids CMCs, Cardiac mesoderm cells, CO: cardiac organoid
